# Environmental and Psychosocial Interventions in Age-Friendly Communities and Active Ageing: A Systematic Review

**DOI:** 10.3390/ijerph17228305

**Published:** 2020-11-10

**Authors:** Diego Sánchez-González, Fermina Rojo-Pérez, Vicente Rodríguez-Rodríguez, Gloria Fernández-Mayoralas

**Affiliations:** 1 Department of Geography, National Distance Education University (UNED), 28040 Madrid, Spain; 2 Research Group on Ageing (GIE-CSIC), Institute of Economics, Geography and Demography (IEGD), Spanish National Research Council (CSIC), 28037 Madrid, Spain; fermina.rojo@csic.es (F.R.-P.); vicente.rodriguez@csic.es (V.R.-R.); gloria.fernandezmayoralas@csic.es (G.F.-M.); 3 Ageing Network of the Latin American Population Association (ALAP), Rio de Janeiro, Brazil

**Keywords:** age-friendly cities and communities, active ageing, intervention, systematic review, PRISMA guidelines, quality of life, environmental gerontology

## Abstract

*Background:* The academic literature contains little information regarding the interventions that create age-friendly cities and communities in order to promote active ageing. *Objectives:* A systematic review was carried out to determine the available empirical evidence in relation to the characteristics, content and effectiveness of interventions aimed at improving environmental and psychosocial risk factors for older people, from the perspective of age-friendly communities and the promotion of active ageing. *Methods:* Following the Preferred Reporting Items for Systematic Reviews and Meta-Analyses (PRISMA) guidelines, the studies retained in this review were identified through a systematic search of the academic literature in selected electronic databases including Web of Science and Scopus. Independent critical appraisal and data extraction were conducted by two reviewers. The checklist was used to assess the quality of the articles. *Findings*: The search identified 1020 potentially eligible documents, of which 11 satisfied the established criteria. Non-exhaustive practices prevailed over rigorous investigations, with a high proportion of studies observed to be of low methodological quality and at high risk of bias. This reflected the predominance of uncontrolled interventions. Environmental interventions were focused on reducing risk and adapting the everyday environmental setting, while psychosocial interventions prioritised social strategies (behavioural changes, promotion of participation) and training. Interventions were more effective in certain domains of age-friendly cities and communities such as transportation and housing, followed by increased participation as a lifestyle-related behavioural change. The inferred changes were associated with providing information and enhancing skills; modifying access, barriers, exposures, and opportunities; enhancing services and support; continuity and effectiveness of changes over time; and modifying policies based on the bottom-up approach of age-friendly cities and communities (AFCC). *Discussion and conclusion:* Interventions focused on personal and organisational aspects might have positive effects in the longer term. However, fewer changes would be observed in interventions revolving around changing lifestyles owing to the impact of complex multi-causal factors. The relative effectiveness in terms of health calls into question the design of interventions and the supposed “friendliness” of certain communities. There is a need to encourage sound longitudinal research aimed at providing key knowledge for the implementation and evaluation of public policies, and to encourage age-friendly community programmes to promote active ageing.

## 1. Introduction: Background and Current Situation

### 1.1. Active and Healthy Ageing and Age-Friendly Cities

Society has evolved to produce populations with greater longevity, resulting in demographic ageing, and this represents an extraordinary achievement at the same time as it poses a social challenge. This process has gained consistency in the transition from the last century to the present one, with the United Nations taking note of the inevitable and widespread move toward an aged demographic structure.

In the context of this foreseeable scenario, the United Nations organised the First World Assembly on Ageing in Vienna in 1982, at which the foundations were laid for the subsequent development of general and specific ageing policies. Recommendations 19 to 24 of the final report from this world assembly (the Vienna International Plan of Action on Ageing, or VIPAA) highlight attention to health in a setting that facilitates older people living independently in their communities for as long as possible, through policies to improve housing and the built environment [1]. These ideas remained part of the discussion held during the Second World Assembly on Ageing, which took place in 2002. The Second Assembly saw the adoption of the Madrid International Plan of Action on Ageing (MIPAA), which addressed the challenge of constructing a society for all ages, focusing on three priority directions, one of which sought to secure supportive environments for older people to promote independence and empower older persons with disabilities to participate fully in all aspects of society [2]. The other directions concerned older persons and development, and advancing health so as to promote wellbeing and quality of life.

As part of the increasing awareness of the challenge of ageing, other paradigms developed in the context of the United Nations for the recognition of the social role played by older people. Perhaps the most important, in the early years of the twenty-first century, is the paradigm of active ageing (AA), which the World Health Organisation (WHO) contributed to the Second United Nations World Assembly on Ageing. From a lifelong perspective, AA places an emphasis on optimising opportunities for health, participation and security in order to enhance quality of life as people age, and this concept has been revised and expanded to incorporate the fourth pillar, namely lifelong learning [3]. This optimisation process must be approached based on a series of factors, among which physical and social setting occupy a prominent position for decision-makers at all levels, across all sectors [3]. The WHO has recently changed its focus to push more decisively for healthy ageing that encompasses the components of AA to a large degree, stressing as part of its strategy the role played by environments that are supportive for older people in different areas (health, long-term care, transportation, housing, work, social protection, information and communication). This change is implemented in the WHO’s Decade of Healthy Ageing [4], in which environment plays a key role in maintaining the functional ability of older people or in facilitating comprehensive attention and healthcare.

The components of AA are also embedded as a framework for the Global Age-Friendly Cities project [5], and 2010 saw the creation of the Global Network of Age-Friendly Cities and Communities (GN-AFCC), with the mission of developing more age-friendly cities worldwide (https://extranet.who.int/agefriendlyworld/who-network/). Developing age-friendly environments is also one of the strategic objectives of the WHO’s Global Strategy and Action Plan on Ageing and Health [6]. Age-friendly environments help to foster healthy ageing in two ways: by supporting the building and maintenance of intrinsic capacity across the life course, and by enabling greater functional ability so that people with varying levels of capacity can do the things they value ([6], p. 10). Moreover, objective five of the aforementioned global strategy is focused on improving measurement, monitoring and research on healthy ageing, including identifying the attributes of an age-friendly environment and what interventions work to create more age-friendly environments [6].

The GN-AFCC directs its priorities at eight areas established following investigation of the experience of older people, caregivers and service providers: housing, transportation, information and communication, outdoor spaces and buildings, community support and health services, social participation, civic participation and employment, and respect and social inclusion. In these areas, starting from a baseline profile, cities and communities can monitor progress and assess the effectiveness of their age-friendly actions [7], for which purpose they need to develop tools according to domain (of functional ability), process (planning, etc.), thematic priorities (such as ageism, elder abuse, and reducing care dependency), regional context, and/or priority area ([7], p. 22).

Together with the evaluation of the progress of ageing in the Decade of Healthy Ageing (2020–2030), the GN-AFCC is also aligned with the 2030 Agenda and its 17 Sustainable Development Goals (SDG), which include the commitment to ensure that all human beings can fulfil their potential in dignity and equality and in a healthy environment [8]. In particular, SDG 11 (sustainable cities and communities) focuses on making cities and human settlements inclusive, safe, resilient and sustainable. Annual SDG reports ensure that these goals are monitored, but the indicators have been seriously affected in 2020 by the impact of the COVID-19 pandemic (https://unstats.un.org/sdgs/report/2020/goal-11/).

The main action principles that are included in these ageing policy documents recognise the role of measures and programmes that pursue interventions to improve living conditions for older people and lobby nations to use resources to that end. For example, the third assessment of the MIPAA in Europe saw several countries reporting having taken measures to improve the physical and social environment in which older people live, at various levels [9]. For its part, the Decade of Healthy Ageing also provides for results to be measured using indicators including one that examines the adaptation of environments to people [4]. However, such measures are not often evaluated with the purpose of assessing their usefulness, at least at this macro level; this assessment is far more feasible at local or infra-local scales.

In this context of growing interest in active and healthy ageing, it is critical to develop research regarding age-friendly cities and communities (hereinafter, AFCC) in order to identify possible knowledge gaps.

### 1.2. Summarising Existing Knowledge Regarding Age-Friendly Cities and Communities

Based on the seminal AFCC model established by the World Health Organization (WHO) in 2006 [5], there has been a great deal of study of AFCC and there is extensive academic literature across various disciplinary fields [10], with the last 10 years representing the most prolific period [11,12,13], especially in Canada, the United States of America, Europe and Hong Kong [14]. The contribution of academic studies regarding developing countries remains marginal [13].

In line with the growth of the WHO GN-AFCC, local institutions in particular have addressed the issue of adjusting cities and communities to make them age-friendly for their residents, in order to promote active and healthy ageing and therefore improve quality of life and wellbeing. This has been reflected in publications mostly produced in the form of reports on results, with few having been subject to peer review. In the context of academic literature on AFCC, literature reviews have been carried out that indicate research trends on the topic. In line with their conceptualisation, these reviews approach AFCC from various perspectives, whether global or domain-specific.

From a global perspective, the features of urban age-friendly environments based on action frameworks have been identified in a review that examines the evidence of approaches and interventions to make cities places that promote the independence of older people [15]. The AFCC model has been applied not only to cities, but also to communities, states, businesses, universities, and public health and healthcare systems, demonstrating a need to create synergies among the different AFCC initiatives under the umbrella of the global age-friendly ecosystem [12]. Less attention has been paid to the study of factors related to the age-friendliness of environments in rural communities [16]. Other reviewed topics have included terminology to describe the age-friendly environment [13,15,17], implementation and development models, challenges and opportunities [13], dimensions of the physical and social environment and policy and governance factors leading to the definition and application of this action framework [17], policies related to AFCC and their impact on wellbeing and quality of life [14], factors that act as facilitators or barriers in terms of the implementation of AFCC initiatives [10], and the need for adaptation of environments and particularly for specialist housing for older people [18].

Various reviews have been carried out using a domain-specific approach, including social isolation and intervention to meet the needs of the older population and minimise the effects of loss of contacts, friends and family members, reduction in mobility and loneliness in studies in Manchester [19] and China [20], adjusting the environment to its residents under the person–environment fit principle, health/disability/falls, and other topics associated with housing programmes and initiatives [21], the walkability, accessibility and safety of the built environment [22], provision of long-term social and healthcare services through the use of mobile devices (eHealth) [23] and the promotion of healthy ageing [24], housing quality standards and habitability to facilitate ageing at home [25], and the factors influencing the fostering of respect and social inclusion and their impact on physical and psychological health and wellbeing [26].

For specific groups of older people, the methodology of age-friendly environments has also been subject to literature reviews focusing on the population with dementia living in long-term care facilities, to assess the effectiveness of the design of open and natural environments in improving the symptoms of dementia [27], and on older immigrants in community dwellings in terms of the factors that influence social inclusion [28].

In the context of various ageing initiatives, especially the WHO’s Age-Friendly Cities [5], the MIPAA [2], and the SDG [8], Mihnovits and Nisos described a need for indicators to measure the suitability and habitability of housing for older people [29]. This need to evaluate the age-friendliness of environments has also been observed at a more global level [30]. In relation to the spread and acceptance of the AFCC paradigm worldwide, Dellamora et al. carried out a scoping review to identify reliable questionnaires and indicators related to age-friendliness for researchers, public policymakers and community leaders to use in order to measure progress in developing age-friendly environments [31]. Reference has also been made to a need to adopt new methods and instruments, collaborative research and country-based comparisons, as well as for older people to participate in the development of age-friendly environments [13].

A case study in the British city of Liverpool has highlighted the complexity of designing and developing an evidence-based evaluation tool to assess age-friendly initiatives [32]. A comparative analysis of Manchester and Brussels concluded that both cities share a series of key factors as regards strategies to foster age-friendly environments, and that these factors may be significant in terms of applicability to other cities, particularly those related to the inclusion of ageing issues in social policies and the integration and participation of the older population in driving the age-friendly agenda [33].

This summary of AFCC reviews has not found any studies that examine the topic of intervention or experimental design of age-friendly environments for the older population, except for the impact of interventions on health and wellbeing to foster respect and social inclusion [26].

### 1.3. Approaches to Interventions

The academic literature reports various attempts to define intervention. However, there is a prevailing absence of consensus owing to the complexity of multiple converging factors. Intervention has a social utility; it is aimed at mitigating or preventing situations of individual, social and environmental risk, as well as contributing to the implementation of actions intended to resolve specific problems affecting individuals, groups and communities. The various forms of intervention can be classified in two major groups: environmental and psychosocial [34].

Environmental interventions are focused on incorporating adaptations and changes to the physical and social environment, as well as altering individual behaviour in terms of how people negotiate and interact with the environment, through management, organisational change and decision-making [35]. Meanwhile, psychosocial interventions are processes that are precisely designed, planned and executed in order to influence the personal and community wellbeing of the population, by means of changes in values, policies, programmes, allocation of resources, power differentials and cultural norms [36].

A range of intervention-based studies have been carried out in recent decades [20,25,26] with the aim of facilitating the production of theoretical models, such as the AFCC paradigm and the pillars of AA. The purpose of these interventions is to transform a particular social and environmental reality (urban and rural) by predicting and changing people’s social behaviour, as well as altering damaging aspects of their environment [37]. Theoretical and methodological contributions have been made from the interdisciplinary perspective of environmental gerontology, with the objective of identifying, analysing, changing and optimising the relationship between the ageing person and their physical and social environment [38]. Specifically, the ecological model of competence [39] has acted as a theoretical reference point in the study of AFCC, to explain how conduct is a function of individual competence (physical health, sensory and perceptual capacities, motricity and cognitive capacity) and the environmental pressures to which the individual is exposed in their community (architectural barriers, violence) [40]. Older people can negotiate with the physical and social environment (housing, neighbourhood), trying to find a balance between their capacities (progressively diminishing with age) and environmental pressures on different geographical scales [41,42]. In addition, older people have been identified as having heterogeneous capacities to adapt to day-to-day environments, where spatial experience, identity and attachment to place play significant roles [43].

The effects of an individual’s social and spatial context, such as their housing, neighbourhood and city, can potentially affect individual outcomes during ageing and on various scales [44]. Certain non-age-friendly interventions in urban communities can discourage healthy lifestyles and aggravate the risks of social exclusion and relocation for more vulnerable older people [45]. Designing AFCC therefore involves understanding the positive and negative experiences of older people in relation to their daily environment (urban and rural), from objective and subjective perspectives, analysing habitability and environmental inequalities [46].

The academic literature shows limited theoretical understanding and empirical evidence regarding which environmental features and social resources make communities more age-friendly for older people and promote AA [15]. This circumstance has been aggravated by the scarcity of assessments of the benefits of intervention programmes aimed at creating age-friendly cities [30], which has constrained the progress of public policy in this area [47].

In this paper, intervention (environmental and psychosocial) is understood as the range of strategies that are planned and aimed at understanding, predicting, changing and resolving a practical problem of psychological, social and environmental origin at individual and community levels, with the purpose of encouraging more age-friendly communities and promoting AA, and thereby improving quality of life among the older population.

Following a systematic review procedure, the main goal of this article is to identify the available empirical evidence regarding interventions in the field of AFCC that are intended to promote active and healthy ageing. The specific goals are to analyse the features, content and effectiveness of interventions aimed at improving environmental and psychosocial risk factors.

## 2. Material and Methods

This study forms part of a broader literature review that is intended to update the knowledge of academic literature regarding AFCC across all territorial contexts, whether urban or rural. Thus, this paper supplements the review carried out in another article published in September 2014, which excluded rural areas [15]. The first step in this broad study was to carry out a systematic review as part of the search, location, assessment, extraction and analysis of useful information. The second phase, to address the specific aim of this paper, was to scrutinise and analyse only those documents that examined the empirical evidence concerning interventions related to age-friendly communities. The intention was to compile relevant information according to specific criteria, by means of a systematic and reproducible search to identify all the studies that satisfied the criteria for eligibility, assessment of validity and summary of results [48].

This partial approach did not include a meta-analysis, owing to the assumption of heterogeneity of the potential studies of interest.

The Preferred Reporting Items for Systematic Reviews and Meta-Analyses (PRISMA) key principles were followed for the systematic review [49,50]. The PRISMA statement was adopted because it is also used in systematic reviews of other fields and other types of research, and particularly in assessments of interventions, in addition to being used in the reporting of results that evaluate randomised trials in clinical practice.

### 2.1. Search Strategy: Data Sources and Search Criteria

Web of Science (WoS) and Scopus were chosen as the literature platforms, due to their position as two of the leading databases worldwide [51,52]. They are among the most extensive databases [53], behind Google Scholar, although the latter has a lower level of quality control [54] and the advantages of its coverage tend to be for low-impact documents published in non-scientific journal sources [55]. For the purposes of this study, the WoS databases used were Core Collection, MedLine and Scielo.

In order to identify the relevant literature regarding applied or theoretical research into AFCC, and based on the common bibliographical references for the topic, the search was implemented using the fields of title, abstract and keywords, including the term “age-friendly”, in order to obtain all of its possible appearances from both a global and a domain-specific perspective (in its eight domains: outdoor spaces and building; transportation; housing; social participation; respect and social inclusion; civic participation and employment; communication and information; and community support and health services). The term “age-friendly” was combined with other possible synonyms, plural forms and different spellings (aging, ageing, elderly, city/ies, community/ies, environment, neighborhood/neighbourhood, rural, urban). The search syntax for both platforms is shown in Table 1. The use of this search term is based on the seminal conception of the paradigm [5] and on its extensive use by the project leaders in Canada. Other terms are also used in the literature, such as “liveable community” and “lifetime neighbourhood”, with these terms particularly popular in the United States and in the United Kingdom, respectively [17]. In any case, all the terms share the common denominator of the original concept [13].

The other search criteria used were date, type of document and language. The publication date was set between 01-01-2007 and mid-2020 (specifically, 25-03-2020 for WoS and 16-06-20 for Scopus, both of which were the date before that on which the search was performed). The starting time corresponded to the year of publication of the global action framework “Global Age-Friendly Cities: A guide” [5]. Types of documents were limited to journal articles, reviews, monographic works and edited books/chapter of edited books, as part of the search for high-quality peer-reviewed publications. The languages searched were Spanish, English, French and Portuguese.

### 2.2. Study Selection and Quality Assessment

**Figure 1:**
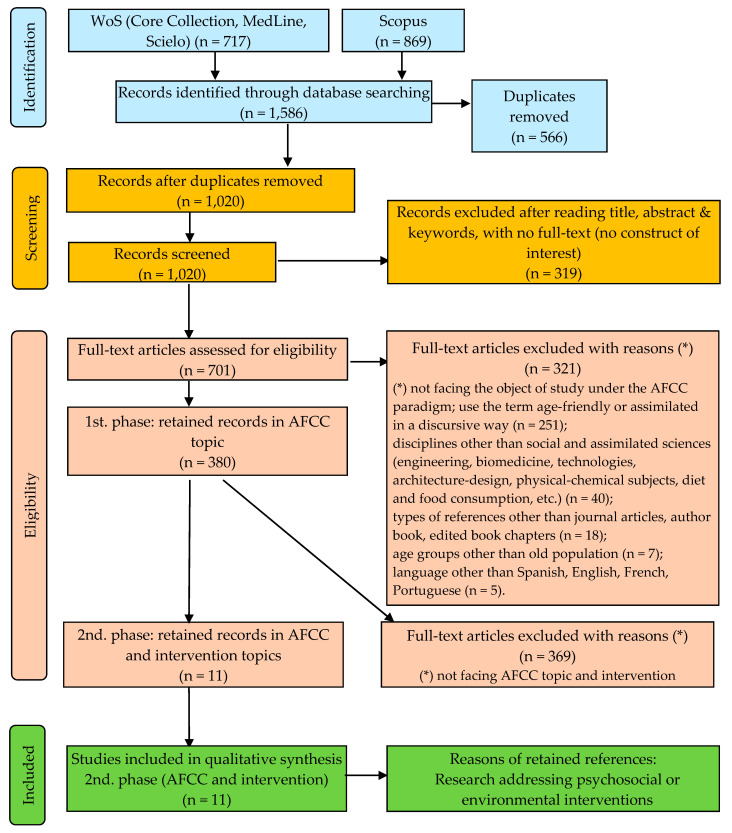
Flow of information through the different phases of the systematic review. WoS: Web of Science; AFCC: age-friendly cities and communities.

A total of 1586 references were obtained (717 from WoS and 869 from Scopus), which were reviewed for suitability and quality as regards the study aims of using the method of independent critical evaluation and data extraction by two reviewers. The documents were initially classified as related to AFCC, unrelated or uncertain. Those classified as unrelated were eliminated from subsequent phases of analysis. Disagreements were resolved through discussions with the review team as to the inclusion or exclusion of uncertain documents until a consensus was achieved. The publication search and selection flowchart is shown as Figure 1.

The elimination of duplications, using the EndNote reference manager and direct observation, resulted in an initial exclusion of 566 references, with 1020 records retained. In relation to the thematic inclusion criteria of AFCC-related studies, an initial selection was made by reading the content of the title, abstract and keywords, resulting in the exclusion of 319 documents. The full text of the document was reviewed in the event of doubt, leading to the rejection of a further 321 documents.

The reasons for exclusion included not approaching the topic from the perspective of the AFCC paradigm or an express extension of it, using the term AFCC discursively but without setting aims based on the model, belonging to fields other than social sciences, psychology, social work or social health, and using languages other than Spanish, English, French or Portuguese. Types of documents such as editorials, commentaries, letters to the editor and complete edited books (only chapters of edited books were retained) were also excluded. As such, only original studies that approached the topic from a theoretical or applied perspective and used quantitative, qualitative or combined methods were retained through the initial review, screening and eligibility phase.

### 2.3. Eligibility Criteria

A second selection and quality-control phase involved scrutinising the references retained from the first phase to compile the studies of AFCC and psychosocial or environmental interventions, intended to build age-friendliness into environments by adapting and modifying those environments to the population experiencing the ageing process. Ultimately, 11 references regarding AFCC and intervention were retained, regardless of the type of intervention and how it was managed, which constitutes the study aim of this article.

Original intervention-based research was selected during this review. Various types of studies were included: (i) non-random and random controlled interventions (participants selected at random and a control or comparison group subject to standard practice or no intervention); (ii) non-controlled interventions (without a control group), including cohort studies (pre-post), and interrupted time series studies; and (iii) crossover interventions (where subjects receive two or more separate interventions for a period of time and act as their own control).

The types of participant were older people and professionals involved in their care. In publications with mixed participant groups, studies in which older people made up at least 50% of the target population were included.

The review incorporated publications from any country or geographical scale: macro (metropolitan, urban, rural), meso (intra-urban, neighbourhood) and micro (housing, building, public space).

With regard to intervention type, the review included environmental and psychosocial interventions, solo or combined, aimed at improving any risk factor for older people based on an approach focused on AFCC and the promotion of AA. Environmental interventions incorporated adaptations and changes to the physical and social domains/aspects [35]. These interventions included: modifications of the daily environment (changes to remove barriers and improve function), assistive technology (devices), information/education, and risk reduction strategies (assessment, awareness-raising, collective problem solving, context adaptations, significant assistive technologies and behavioural safety strategies) [56].

Psychosocial interventions are intended to understand, predict and change people’s social behaviour, as well as to modify harmful aspects of their environment, with the aim of improving quality of life [37]. From this perspective it is possible to distinguish various kinds of intervention: psycho-educational, psycho-therapeutic, social, educational, multi-component and support groups [57]. The selected studies implemented assessments of results related to behavioural changes in terms of lifestyle, such as improvements in health knowledge and behaviour, morbidity and mortality risk factor indices, changes in organisation results (factors related to work, safety and patient care), and changes in outcomes for the eight AFCC domains: outdoor spaces and building; transportation; housing; social participation; respect and social inclusion; civic participation and employment; communication and information; and community support and health services.

### 2.4. Data Collection and Risk of Bias

The data were independently extracted by two reviewers, who then verified the extracted data and discussed inconsistencies until they reached a consensus. The table displaying the data includes details of each study relating to the participants, environment, intervention and measures of results (Table 2, Table 3 and Table 4).

In addition, the PRISMA statement and the Cochrane handbook were used as tools for assessing quality and type and degree of risk of bias (selection, performance, detection, attrition and reporting) in the studies retained [49,50,58].

### 2.5. Summary Method

The studies were summarised in narrative form, which is an appropriate method to assess design data from heterogeneous studies [59]. This narrative summary facilitates an explanation of the findings of the selected studies, structured based on the characteristics, content and effectiveness of the interventions (Table 2, Table 3 and Table 4).

## 3. Results

### 3.1. Studies Included

The initial search produced 1586 potential documents. The successive title and abstract review phases reduced the sample to 1020 records, and subsequently to 380, with 640 potential manuscripts excluded. The full texts of the remaining 380 manuscripts were reviewed based on the inclusion and exclusion criteria. As a result of this process, 11 studies were included in this review [60,61,62,63,64,65,66,67,68,69,70] (Figure 1).

The specialist fields of the retained publications’ authors were dominated by multidisciplinary approaches (54.5%), such as nursing and architecture [64], architecture, geography and psychology [70], and nursing and innovation science [69], followed by health sciences (nursing, psychology, occupational therapy, sport sciences) [62,68] and social sciences (political sciences, social work, sociology) [60]. To a lesser degree, studies were identified in specific fields of social sciences (27.3%), such as social work [65,67] and geography [61], and health sciences (18.2%), such as nursing [66] and psychiatry [63].

Based on the paradigm of AFCC [5,7], the theoretical approach of AA [3,71,72,73,74] was referred to in 54.5% of studies [60,61,65,66,68,70], and the approach of healthy ageing [4] was referred to in 54.5% of cases [60,61,63,66,68,69]. Additionally, in 36.4% of retained publications [60,61,66,68] the theoretical approaches of AA and healthy ageing were cited together [75]. However, in other studies AA was ignored (45.5%) [62,63,64,67,69], and healthy ageing was not mentioned (45.5%) [62,64,65,67,70], and in 27.3% of cases both approaches were omitted [62,64,67].

The theoretical background to the selected studies focused on the important relationship between the characteristics of the physical and social environment of communities (that are intended to be age-friendly) and active and healthy ageing. From the perspective of environmental gerontology [76], emphasis is placed on the theoretical assumptions of the ecological model of ageing [39], which is cited in 27.3% of manuscripts [60,61,70]. Despite their importance, these theoretical assumptions were omitted, or lightly referred, to in almost two out of every three studies [62,63,64,65,66,68,69].

The included publications mainly used designs based on uncontrolled interventions (pre-post cohort studies, time series) (45.5%) [60,61,64,65,67], and random and non-random crossover interventions (pre-post studies with a control group, where each subject receives two or more separate interventions for a period of time) (36.4%) [63,66,68,69], and to a lesser extent, non-random controlled interventions involving the inclusion of comparison groups (users and non-users) (18.2%) [62,70] (Table 2).

The designs of the studies included were shaped to a large extent by the human and financial resources available for interventions, responsibility for which lay with universities and research centres (63.6%) [60,63,64,67,68,69,70], public authorities (45.5%) [60,61,62,68,70], non-governmental organisations (18.2%) [60,65] and the private sector (9.1%) [66]. One study was carried out in part thanks to help from non-governmental organisations, but with no other financial support [65].

### 3.2. Sample Characteristics

Sample sizes varied between three and 1683 participants (total participants: 3812; median: 96) (Table 2). In 81.8% of studies, the participants were non-institutionalised older people [60,61,62,63,64,65,67,69,70], while only one study included institutionalised and non-institutionalised older people as participants [68]. The 36.4% of studies involved the participation of professionals (hospital staff, carers, planners) [61,64,66,69] and, to a lesser degree, family members [64,66].

The main criteria for inclusion of older people as participants were age (90.9%) [60,61,62,63,64,65,67,68,69,70], gender (90.9%) [60,61,62,63,64,66,67,68,69,70], functional and cognitive level (45.5%) [62,64,67,68,69], medication self-management (9.1%) [69] and work status (9.1%) [66]. In 63.6% of retained publications, participants were aged 60 years and older [62,64,65,67,68,69,70], with 18.2% of cases involving participants aged 50 and older [60,63] and one study that did not specify the older people’s age [61].

The average age of the older adult participants in the retained publications was 76.1 years, with 39.2 years for professional participants [60,62,66,68,70]. However, 54.5% of studies did not provide these data [61,63,64,65,67,69] (Table 2). Additionally, participant gender was described in detail in seven studies [60,62,63,67,68,69,70], where women represented an average of 71.9% of participants (as opposed to 28.1% being men). The other studies did not include sufficient information on gender [61,64,65,66]. It is striking that none of the retained publications specifically adopted a gender perspective.

In relation to the geographical location of the interventions, studies performed with participants resident in Asia stood out (Table 2), including Hong Kong (18.2%) [60,65], Taiwan (9.1%) [66] and Thailand (9.1%) [64], followed by Oceania (Australia) [61,62,67], North America (Canada) [63,68], and Europe, including the United Kingdom [70] and Sweden [69]. No studies in Latin America, the Caribbean or Africa were identified in the review.

Participant selection was non-random in 72.7% of studies, which used the snowball or convenience procedure [60,61,62,64,65,69,70], while the others opted for random participant selection [66,68], and a single study did not provide sufficient data regarding the participant selection process [67] (Table 2).

### 3.3. Intervention Characteristics

From the perspective of implementation, all studies selected non-pharmacological interventions and a combination of different types of strategies with distinct conceptual bases. In fact, the multicomponent interventions were notable for involving a combination of environmental and psychosocial intervention strategies (45.5%) [63,64,68,69,70], followed by strategies specifically focused on environmental (36.4%) [60,61,62,67] and psychosocial interventions (18.2%) [65,66] (Table 2). The environmental interventions identified included various strategies [56], including those focused on risk reduction (assessment, awareness-raising, collective problem solving, environmental adaptations, significant assistive technologies and environmental and behavioural safety strategies) (72.7%) [60,61,62,64,67,68,69,70], adaptations of the daily environment (removal of barriers and improved function) (36.4%) [60,67,69,70], incorporation of assistive technology (devices) (9.1%) [69], and information and education (9.1%) [63]. Additionally, the psychosocial interventions observed adopted various strategies [57], including social (behavioural changes in terms of activity aimed at fostering wellbeing, promotion of social participation and empowerment) (36.4%) [62,65,68,70], training (learning skills) (18.2%) [66,69], psycho-educational (9.1%) [63] and support groups (9.1%) [64].

The WHO basis for AFCC was contemplated in the design of interventions in 72.7% of studies [60,61,62,63,65,67,68,70] (Table 2). The remaining studies involved adaptations related to the AFCC aims, including proposed models for friendly and safe housing (risk of falls, healthcare technology in the home) for older people [64,69], and for age-friendly hospitals [66]. Intervention strategies were also implemented focused on a single AFCC domain (45.5%) [62,65,67,69,70], and others addressed several domains at once (housing, transportation, social and health services, civic participation and employment, social participation, and respect and social inclusion) (36.4%) [63,64,66,68] and, to a lesser degree, interventions tackling all of the AFCC domains (18.2%) [60,61]. The AFCC domains receiving most attention were: transportation (54.5%) [60,61,62,65,67], respect and social inclusion (45.5%) [60,61,63,66,68], housing (36.4%) [60,61,64,68], community support and health services (36.4%) [60,61,66,69], social participation (36.4%) [60,61,63,68], outdoor spaces and buildings (27.3%) [60,61,70], civic participation and employment (27.3%) [60,61,64], and communication and information (18.2%) [60]. Domains associated with the physical environment dominated among environmental interventions (90.9%), including transportation, housing, outdoor spaces and buildings, and community support and health services. Psychosocial interventions opted for domains focused on social and personal context (54.5%), including social participation, respect and social inclusion, and civic participation and employment.

With relation to the format of interventions, 45.5% were individually-based [60,62,67,68,70], with 36.4% in group format [61,63,64,65], and both formats were combined in 18.2% of cases [66,69] (Table 2).

Interventions were monitored and lasted for between 20 h [68] and 30 months [70] (average: 10 months; median: 4 months), but two studies did not provide sufficient data regarding duration [64,65]. Ten studies evaluated effects immediately upon completion of the intervention (short-term), and only one study evaluated effects in the medium term (between 3 and 6 months later) [70] (Table 2).

The intervention settings for the retained studies were principally on the macro geographical scale of urban and metropolitan communities (45.5%) [60,61,62,67,69], with fewer approaches jointly examining urban and rural communities (9.1%) [63], the meso scale of intra-urban communities (districts, neighbourhoods) [64,65,68,70], and the micro scale (hospitals) [66] (Table 2). No study specifically focused on rural communities.

### 3.4. Strategies, Instruments and Measured Used

In the interventions studied, preventive strategies were observed in 81.8% of cases [60,61,62,63,64,65,67,68,69,70] (Table 2). These were aimed at reducing the scale of environmental and psychosocial risk, and included making adjustments to the physical environment (removal of barriers, replacement of furniture) and social environment (changing the functions of members of the community) [65]. Additionally, management strategies were also identified (63.6%) [60,62,63,64,66,69,70], being designed to improve the resistance of those exposed to environmental and psychosocial risk by means of combined actions over time (training activities) that act on the sources of stress [66]. However, no therapeutic strategies were noted aimed at providing medical and/or psychological (telecare services) treatment to those affected by stress or other manifestations of risk (cases of violence, abuse).

The methodological strategies implemented in appraisals of interventions included selected studies that used mixed (quantitative–qualitative) approaches (54.5%) [61,63,65,66,67,69] (Table 2), with a combination of semi-structured questionnaires, focus groups, workshops, SWOT (strengths, weaknesses, opportunities, and threats) analysis and observation via participative design (participative research-action). These were followed by quantitative approaches (27.3%) [60,62,70], such as pre- and post-intervention structured questionnaires, and accelerometry (direct measurement of physical activity). Qualitative approaches were applied to a lesser extent (18.2%) [64,68], including in-depth interviews, direct observation and focus groups.

Out of the studies retained, 45.5% described instruments and measures aimed at evaluating the improvement of risk factors for physical and psychological health (Table 2), including objective levels of physical activity (accelerometry) [70], self-assessed state of health [60], general scale of health (EQ-5D) [70], adult index of personal wellbeing [69], scales of serenity [69], medication adherence [69], self-management ability (30 elements) [69], satisfaction with life [69], quality of life (CASP-19) [70] and instrumental activities of daily living [70], the personal component of the housing enabler (functional limitations and dependence on mobility devices) [67], and the Thai Falls Risk Assessment test (Thai-FRAT) [64]. However, two studies did not explain in sufficient detail the measures implemented to assess health risks [61,63], and four did not include measures to assess those factors [62,65,66,68].

Behavioural changes in terms of lifestyle were precisely assessed in 27.3% of studies (Table 2), using measures such as the subscale of attitudes towards one’s own ageing [63], the instrument for assessing changes in participation in social activities [62], and a multiscale instrument to assess self-reported frequency, type and location of outdoor activities [70]. However, more than six of every ten did not provide in-depth explanations of measures to assess behavioural changes [60,61,64,65,67,68,69], while one study did not include measures to determine these factors [66].

An assessment of changes in organisations was barely analysed (9.1%) [66] (Table 2), using measures such as the geriatric attitudes scale (GAS) and a model of change at the institutional level (communication and management level). In contrast, the rest did not perform any assessment of these changes.

Variations in outcomes for the AFCC domains were examined in detail in a third of references (Table 2), using measures such as a multiscale instrument to assess age-friendliness of communities for older people [60], a satisfaction questionnaire for older bus users (changes in frequency and ease of use) [62], a scale for assessment of environmental housing risk (architectural barriers) (Thai-FRAT) [64], and the neighbourhood open space (NOS) scale [70]. The measures used to assess these changes were not clearly explained [61,63,65,66,67,68,69] in the remaining studies.

### 3.5. The Core Components and Elements of the Interventions

The retained studies indicated the core components and elements of the heterogeneous interventions subject to analysis. In relation to the component of “providing information and enhancing skills”, public information campaigns were carried out to educate older adults on the problem and how to address it [60,61,70]. Awareness-raising and skill-building workshops also took place [60,62,64,65,66,69], in addition to community support groups [61,63,64], training programmes (walks, physical exercises) [60,64,68], home visits and telephone reminders [64,67,69,70].

For the component of “modifying access, barriers, exposures and opportunities”, strategies were implemented that were aimed at reducing risk to stress factors (architectural barriers) [60,61,62,64,65,67,68,70] that compromise health and safety (falls, psychological problems, dementia) [63,64,68,69,70]. However, it was observed that barriers prevailed over the limited facilitators in public spaces (green spaces, leisure areas, free access) that foster social inclusion and reduce risks to health (dependence) [68,70]. Therefore, strategies implemented and based on proactive conduct notably included planning of trips (outings, activities), physical and preventive support from social networks on outings, and avoidance (avoiding unknown spaces and prioritising activities close to home) [68]. Opportunities were also offered for social participation and integration, to counter age-based stereotypes and discrimination [61,65,66,68]. Specifically, the promotion of social interaction was a facilitator of everyday activity and quality of life in general among older people in the neighbourhood [70].

The “enhancing services and support” component included measures intended to increase the quality of spaces and services (social services, public transport) [60,61,62,65,67,68,70], as well as to improve support (volunteers and professionals) in terms of preventive health actions [63,64,65,66] and emergency situations [61,68]. In fact, a clear link was observed between social commitment and the provision of accessible, affordable and reliable services for older adults [67,69,70]. However, awareness and commitment levels among those responsible for public policy may cause policy shifts, improvements in facilities and services (pedestrian infrastructure, signposting and frequency of public transport) [62,67,70] and changes in the type and degree of participation by older citizens in decisions affecting the community [65]. The knowledge and attitudes of professionals (nurses, social workers), family caregivers and citizens in general regarding older people, may also encourage participation and maximize the benefits of spaces and services [65,66,67,68,69].

In terms of the “continuity and effectiveness of changes over time” component, a degree of ongoing support was provided for promoting health [63], fostering empowerment [65] and access to spaces and services (public spaces, inclusive housing) [60,67,68,69,70]. However, the majority of interventions did not contemplate specific measures for the continuity of future support in terms of preventive health, safety and social inclusion practices by means of continuous training, incentives to purchase inclusive housing and free acces to services (public transport).

Finally, the “modifying policies” component consisted of public policy changes in order to achieve goals, such as designing and implementing policies based on the bottom-up approach of AFCC and AA [60,61,62,65] and aimed at encouraging participation and empowerment among older adults [65]. Emphasis was placed on the importance of fostering collaboration among the various authorities and social agents (NGOs and older adults’ asssociations) [60,61], and on the development of programmes (educational and awareness-raising) on ageing [66] and the promotion of health, implemented by professionals [64,65] and older people [63,69].

### 3.6. Effectiveness of Interventions

All the included studies referred to effectiveness post-intervention in terms of encouraging changes in AFCC domain outcomes, and the success rate was 72.7% (Table 3). However, effectiveness was only described in detail in four studies, with significant positive changes in 75% of them, such as user satisfaction with buses (changes in frequency and ease of use) [62], reduced home environment risk [64], and improved perception of neighbourhood open spaces (accessibility, safety) [70]. Relative changes occurred in 25% of cases, with a partial improvement in age-friendliness for five of the eight AFCC domains [60]. Seven articles did not describe any potential changes in sufficient detail [61,63,65,66,67,68,69]. Notwithstanding this, an improvement was reported for the domains of social participation [61,63,68], transportation (accessibility, ease of use) [61,65] and community support and health service [66], as well as a moderate effect associated with housing (technological systems in the home) and transportation services [67,69]. Additionally, as a result of the effectiveness of interventions aimed at bringing about changes in AFCC domains, in 81.8% of studies [60,61,63,65,66,67,68,69,70] one or more pillars of AA were promoted, such as health, safety, participation and continuous learning [77,78].

The effectiveness post-intervention in terms of improving physical and psychological health risk factors was considered in 63.6% of studies [60,61,63,64,67,69,70], with a success rate of 42.8% [61,63,64] (Table 3). In this respect, only five articles described effectiveness in detail, of which 80% reported that there were no significant positive changes in terms of improvement of participants’ physical and self-assessed health [60,67], personal wellbeing, life satisfaction, quality of life, levels of activity or medication adherence [69,70]. To the contrary, other studies referred to positive changes post-intervention, including lower frequency of falls [64]. In addition, positive changes in health post-intervention were reported in 18.2% of studies that did not describe effectiveness in detail [61,63].

Except for one study [66], there were references to post-intervention effectiveness of actions aimed at encouraging positive behavioural changes to lifestyle, with a success rate of 70% (Table 3). However, only three studies provided a detailed description of any potential behavioural changes, focusing on participation in social and outdoor activities as well as on the use of services (public transportation) [62,70], and attitudes toward one’s own ageing [63].

A positive effect was reported in terms of the attitude of hospital employees towards ageing. Effectiveness post-intervention in terms of producing changes in organisations thus achieved a success rate of 100% [66] (Table 3).

Unintended or unexpected negative effects of interventions were reported in 36.4% of studies. Participant attitudes affected the potential effectiveness of technological systems in the home [69]; there was no improvement in health or increase in the frequency of use of transportation associated with perceived progress in terms of its age-friendliness [62,67] and health and activity levels did not increase in association with perceived improvements in neighbourhood open spaces (accessibility, safety) [70]. To the contrary, unintended or unexpected positive effects of intervention were observed in 18.2% of studies, including higher social participation [68] and communication [64].

### 3.7. Dropout Rates

Intervention participant dropout rates were reported in 45.5% of studies [63,66,67,68,70], while the remainder did not provide clear information in this respect [60,61,62,64,65,69] (Table 4). Among the studies that did provide information, the dropout rate ranged between 0% [68] and 53.2% [70] (average: 38.4%; median: 51.5%; standard deviation: 20.5%). Nevertheless, none of the studies performed a detailed analysis of the possible reasons for dropout rates (lack of interest, fatigue, illness).

### 3.8. Type and Degree of Risk of Biased Results

In assessing the type of bias risk, following the Cochrane handbook [79], studies based on controlled interventions and crossover interventions (Table 4) were observed to present some kind of bias: (i) performance (non-random allocation of sample) [62,63,69,70]; (ii) reporting (selective reporting of results) [63,68,69]; (iii) attrition (systematic differences in study dropouts) [63,66,70]; (iv) selection (insufficient information regarding initial characteristics of participants) [63,66]; and (v) detection (systematic differences in how results were determined) [68]. In addition, the fact that five studies were based on non-controlled intervention designs [60,61,64,65,67] affected the overall assessment of type of bias risk, since for those studies it was not possible to show potential biases (masking, random allocation) associated with comparison between groups.

Across all of the retained studies, a high level of bias risk was observed related to the potential absence of randomness in participant selection [60,61,62,63,64,65,67,69] (Table 4), insufficient information regarding participant characteristics [61,64,65,67], insufficient information regarding the instruments and measures implemented [61,64,65,68,69], or the results associated with intervention effectiveness [61,63,64,65,67,68,69].

### 3.9. Assessment of Methodological Quality

Based on the Cochrane Handbook [79], the assessment of methodological quality of the studies analysed showed significant variability. Only two studies achieved high quality in their description of the methods and instruments underpinning their results [66,70]. This review of the academic literature has shown a high proportion of low-quality manuscripts, although a small percentage of research into intervention and AFCC has offered significant contributions to the advancement of knowledge.

## 4. Discussion

The results of this systematic review indicated a growing interest in researching AFCC over the last decade [13]. However, the application of document exclusion criteria revealed a limited approach and a scarcity of available empirical evidence with regard to interventions aimed at improving environmental and psychosocial risk factors from a perspective of AFCC and the promotion of AA [15]. A lack of assessments of AFCC intervention programmes has in turn restricted progress in terms of public policy on ageing [30,47].

The retained studies were dominated by multidisciplinary and interdisciplinary approaches, among which social work, nursing, psychiatry and geography stood out. In this respect, various studies [40,80] have noted this importance in studies of interventions based on the AFCC framework and the promotion of active and healthy ageing, since a range of environmental and psychosocial indicators are involved.

The academic literature shows that a lack of consensus as to the conceptual and methodological definition of interventions has affected the viability of broader practical implementation and the potential benefits of interventions [26,34]. In this respect, the retained studies have not specifically contributed to progress in defining the concept of intervention, or to the production of theoretical models under the AFCC and AA paradigm [7,72]. In fact, one third of articles did not refer to the theoretical approaches of active and healthy ageing [62,64,67]. Moreover, a majority of studies omitted or only made passing reference to theoretical positions related to the ecological ageing model [39,81] and to environmental gerontology [40,43]. In this respect, for these studies based on the AFCC framework, the limited theoretical basis provided for understanding the relationship between the characteristics of the physical and social environment of age-friendly communities and the promotion of active and healthy ageing affected the design and, potentially, the effectiveness of the interventions.

Based on the findings of this review, the empirical evidence is verified in relation to the study’s aim. The characteristics and quality of the retained studies, their content and the success or effectiveness of the interventions are therefore focused on reducing environmental and psychosocial risk factors to promote AFCC and active and healthy ageing. The achievement of specific goals is discussed in the following sections.

### 4.1. Discussion: Study Characteristics

The designs of the retained studies showed a preponderance of uncontrolled interventions, followed by crossover interventions. The scarcity of non-random and random controlled designs minimised the potential findings of the interventions, particularly in psychosocial terms, as there was no contemplation of the possibility of monitoring over time and comparing progress (perceived health, functionality) based on control groups. The choice of study design determined the reliability of the effects observed and attributed to the environmental and psychosocial interventions. In this respect, other authors [82] recommend implementing direct comparisons in interventions (random participants and control groups) wherever possible.

These designs were restricted to a considerable extent by the human and financial resources available in the interventions, which depended above all on universities, research centres and public authorities. A lack of funding also appeared among the problems identified in the implementation of the studies [65]. Additionally, a majority of the interventions depended on public calls for proposals and, above all, on local authorities, which affected the design and, in all likelihood, the effectiveness of the programmes. It has been argued that there is a lack of investment in research and that local communities have limited structural and financial capacity to respond to population ageing, particularly in developing countries [46,83]. This complex reality affects the production of empirical evidence and the development of public policies, as well as having the potential to give rise to inequalities between beneficiary communities and those that are excluded [84].

Non-institutionalised older people dominated in terms of sample size, while those institutionalised appeared in only one study [68]. This circumstance reflects a lack of interest in improving the environmental and psychosocial risk factors of the institutionalised population, based on the promotion of interventions from an AFCC perspective where the neighbourhood is an extension of one’s residential care facility. It has been specifically observed that the residents of these facilities benefit from being related to and integrated within the environmental and social context of their neighbourhood (going out for walks in the park, shopping, social relationships), promoting their AA [85]. The age-friendly discourse also runs the risk of excluding other vulnerable groups (dependants, immigrants, LGTB people), restricting the potential of AFCC [86]. In fact, there has been discussion of the influence of economic models on the ideological basis for AFCC, which are in many cases focused on increasing consumption by older people and reducing expenditure related to dependency [87,88].

The majority of the articles considered groups of people aged 60 years and above, with a smaller proportion including those aged 50 and above. However, none of the studies focused on advanced-age cohorts such as nonagenarians and centenarians—groups that are significantly increasing in number worldwide. Significant differences were also observed between age groups in relation to preferences concerning AFCC domains, such as use of public transport, outdoor activity, participation in social activities and housing; this is in line with other studies [89]. Eight studies considered gender as an inclusion criterion. However, none of them specifically examined interventions from the perspective of gender and the AFCC paradigm. This circumstance reflects limited knowledge regarding this perspective. Specifically, it has been argued that it is important to address problems of inequality and social exclusion among older women (access to urban spaces, use of services, job market) and that there is a need to make their reality visible (social participation, physical activity and built environment) in order to contribute to their social inclusion and empowerment [65,90], which is key to developing AFCC [91].

In terms of the geographical location of interventions, most studies were conducted in Asia and Oceania (Australia), followed by North America and Europe. To the contrary, no studies were identified that had been carried out in Latin America, the Caribbean or Africa. This circumstance clashes with the fact that 26% of GN-AFCC members are located in Ibero-American countries (Spain, Latin America and the Caribbean) [7]. The context of this region, with economic austerity, new climate and healthcare challenges (COVID-19), a lack of promotion and pressure from power groups (property speculation), has hampered the expansion and success of this global network [92,93].

Most interventions were implemented in urban and metropolitan communities, reflecting the growing interest in adapting the urbanisation process to an ageing society. However, there is limited knowledge and examination of rural communities [63]. This circumstance is indicative of the focus of age-friendly discourse on cities, justified in part by urban demographic ageing at a global level, and a lack of public policy attention to rural communities, where the aged population is more vulnerable to problems with access to services, social isolation and environmental risks [87]. In fact, an integrative review identified the lack of AFCC-related research performed in rural communities [16].

When examining the characteristics of interventions, prevention and management strategies were observed but not therapeutic ones. The empirical evidence is highlighting the benefits of non-pharmacological treatments based on therapeutic interventions, such as physical exercise and light therapy [94]. Implementation of these types of interventions may have positive effects among older residents, as well as contributing to assessing the success of AFCC.

All studies opted for non-pharmacological interventions, and the majority of cases involved multicomponent approaches based on a combination of various strategies (environmental and psychosocial). Environmental strategies mainly focused on reducing risk and adapting the daily environment, while psychosocial approaches prioritised social strategies (behavioural change, promotion of participation) and education. The majority of articles also followed AFCC guidelines and the remainder made adaptations related to those objectives. In this regard, environmental interventions considered AFCC domains associated with the physical environment, such as transportation and housing, while psychosocial interventions emphasised domains related to social participation and respect and social inclusion. The literature on AFCC is dominated by studies focused on participation as a basis for the design of age-friendly communities, owing to its positive effects on social integration, health and quality of life [86]. AFCCs are driving higher participation and democratic commitment to the rights of older people in cities [95]; nonetheless, some authors maintain that this participation must not become a requirement for all age-friendly initiatives [96]. It has also been argued that certain domains, such as employment and citizen participation (work, volunteering), have been subject to less development and continue to present significant barriers for older people in their communities [97].

The average duration of the interventions was 10 months, with most approaches being longitudinal. In this respect, changes in the attitudes of older participants may involve many months of daily repetition to establish a habit [98]; however, the predominance of interventions implemented for a short period of time (20 h) or on a sporadic basis (once a week or less frequently) have limited their potential positive effects. These circumstances defined the monitoring of participant experiences (fidelity, adherence) and limited understanding of the complexity of the factors analysed and the obtaining of new empirical evidence.

### 4.2. Discussion: Success of Interventions

Post-intervention effectiveness reflected the fact that most studies promoted, at least, one of the pillars of AA. This effectiveness was higher for changes in the outcomes for AFCC domains, followed by positive behavioural changes in terms of lifestyle. However, the lower effectiveness of health-related interventions provides evidence of the possibility that certain initiatives are not particularly age-friendly, which would call into question the design and implementation of certain age-friendliness programmes. The success of interventions and production of findings may also be compromised by differences, sometimes marked, in knowledge and experience among older people, professionals, politicians and researchers regarding age-friendly communities and services. In fact, one example of relative effectiveness has been the partial adoption of assistive technologies, despite the unreceptive attitudes of some older people [69], reflecting the their limited involvement in the identification of needs and adequate solutions [99]. In turn, some authors produce questionable interpretations of their results [60], such as stating that there have been no improvements in participant health but that health-related wellbeing might be improved.

The success of interventions that incorporate personal and organisational aspects may have positive effects in the longer term; this is consistent with other studies [100]. However, fewer changes are observed that are linked to interventions aimed at changing lifestyles, owing to the impact of complex multi-causal factors. In fact, short-term health- or lifestyle-related changes do not tend to have immediate effects on individual wellbeing [59].

In most studies, active social participation, positive social relationships, commitment and inclusion offered an antidote to the stereotypes of ageing. Older people expressed a specific desire to maintain social networks and meaningful personal identities by ageing in place [101]. This new discourse on ageing is therefore redirecting the debate on public policy regarding issues of social inclusion, commitment and community development [17,26], in line with the AFCC promotion.

The framework of AFCC was designed from a down-top perspective, whereby politicians and specialists would exchange efforts and lead age-friendly initiatives, and governance processes would involve older people in the planning of their communities [102]. However, it is unclear what kind of support should prevail when building AFCC. A proactive approach, involving stakeholders in planning and promoting the empowerment of older people to create optimal social and environmental conditions for AA, is not always adopted. In fact, some authors [103,104] have argued in favour of eradicating age discrimination through empowerment, and have criticised an alleged sporadic interest on the part of certain politicians (during election campaigns) in older people actively participating in the design of cities.

Related to change linked with heterogeneous interventions, it was determined by core components and elements such as access to information and enhanced skills, particularly linked to awareness-raising and skill-building workshops. However, a lack of public information campaigns may have limited the success of interventions and the participation of the community. Many of the studies analysed did not address the active role that might have been played by civil society organisations (neighbours, older adults) in the design, implementation and success of interventions. In the majority of the interventions, success may have been affected by the influence of community dynamics. However, there is a lack of evidence regarding the impact of interventions based in communities for the elderly, whose results cannot be generalised at the community level [105]. In this regard, certain interventions based in experimental settings focused on isolated causes (factors including risk of falls, depression and loneliness), without taking into account complex social causes and governance [106].

Changes associated with the removal of barriers that compromise health and safety and access to opportunities for choice and social integration were decisive for the success of interventions. These opportunities must be based on the principles of physical, information-related, social and psychological access. Specifically, the practical implementation of interventions needs to prioritise opportunities for user choice. This would be achieved by means of strategies to facilitate proactive conduct and make support available in the physical and social environment, by offering activities and services that are focused on fostering personal skills and adaptive behaviours [15]. Additionally, the most successful interventions take place in the ordinary environment where the older population spends its daily life [107]. The implementation of interventions in other spaces may minimise the potential benefits of these programmes. Moreover, the environmental context and spatial experience, such as identity and attachment to place, can affect how participants fit into the environment where the intervention takes place [108].

The enhancement of services and support was conditional on their accessibility, reliability and cost, as well as on public spending and the degree of awareness and social commitment of the various community agents (politicians, professionals, relatives). Specifically, citizen awareness and attitudes vis-à-vis older adults can play a more decisive role than local authorities in the success of interventions [86]. In this regard, some studies note that making an environment age-friendly must occur by means of a collective response involving the participation of governments, community organisations, businesses and other private citizens, as well as older people [109]. All of this might offset the austerity of public spending on the promotion of AFCC and AA.

The majority of interventions did not include specific measures such as financial incentives or lifelong training for the continuity of future support. This circumstance favours social exclusion among more vulnerable people (low-income and dependent categories) [60,67]. Some researchers [110,111] have performed a critical analysis of the discourse regarding age-friendliness, warning that it runs the risk of disguising or concealing social vulnerability and inequalities. In this respect, certain “friendly” planning actions may mask gentrification processes, aggravating the vulnerable circumstances faced by older people in terms of social and environmental risks in their daily environment, including social isolation, abandonment and inaccessibility of public services and spaces [112].

Movements towards public policies promoting collaboration between authorities and social agents are decisive in the success of interventions. There is also recognition of the importance of institutional support to guarantee the continuity and success of programmes, as well as of the difficulty of transferring knowledge from local practices to other, more complex urban and socio-cultural systems [65,70].

Some authors [113,114] underline the need to achieve consensus and interdisciplinary complementariness in the design of intervention programmes, based on the fostering of stakeholder communication, environmental optimisation and fit to participant needs and preferences. In turn, the formulation of intervention programmes must be underpinned by a diagnosis of potential participants’ care needs and preferences, as well as their functional, cognitive and sensory capacities, environmental and sociocultural characteristics, the legal and political framework, and training of professionals based on an interdisciplinary approach [33,115]. Moreover, any intervention programme must be subject to regular assessments and open to improvements, in order to guide practice and contribute to community-level decision-making aimed at fostering age-friendliness [32,116].

### 4.3. Discussion: Study Quality

The methodological strategies used in the studies were dominated by mixed quantitative–qualitative approaches. Quantitative approaches notably involved structured questionnaires and direct measurements and qualitative approaches focused on direct observation, in-depth interviews and focus groups. Validated scales were included to measure health [117], quality of life [118], personal competence [119], instrumental activities of daily living, geriatric attitudes [120] and assessment of environmental risk (housing and neighbourhood) [121,122]. Despite this, most studies did not provide sufficiently clear explanations of the assessment measures for the AFCC domains and associated behavioural changes. In this respect, various authors have warned of the persistence of methodological problems [83,123], such as different interpretations of the meanings of the variables and domains analysed by different social and cultural groups. Moreover, key environmental indicators of age-friendly communities were not analysed [15], and some instruments were not included, such as physical environment checklists [124] and other ethnographic methods (photovoice), whose results are offering innovative research possibilities [125].

More than half of studies did not provide clear information on dropout rates, while the remainder reported one in every two participants abandoning the intervention. None of the studies engaged in a detailed analysis of the potential causes of these dropouts. Despite this, it is important to attempt to better identify the risk factors that contribute to dropouts (lack of interest, fatigue, illness) in order to provide key empirical evidence for future research [126]. These causes might shed light on the shortfalls of interventions aimed at older people and provoke changes on the part of those designing ageing-focused public policies.

None of the publications conceptually defined the type of interventions carried out. This significant aspect reflects the observation of a limited theoretical foundation, which has adverse effects on potential results and on the subsequent discussion. The academic literature has included arguments that the AFCC paradigm gives rise to conceptual problems [127]. It has been suggested that the lack of methodological consensus has restricted scientific progress and the obtaining of new empirical evidence [86]. Additionally, the methodological limitations of the implemented interventions show the need for sounder longitudinal studies to identify the characteristics that make a community age-friendly, with priority given to assessing their effectiveness at an individual and a community level, in addition to promoting active and healthy ageing [30].

The review found more non-exhaustive practices than rigorous research. In fact, the methodological evaluation showed a high proportion of low-quality studies in terms of having a high risk of bias, including a lack of randomness in participant selection and insufficient information regarding characteristics, instruments and measures implemented, and the results associated with effectiveness. However, some high-quality studies have made noteworthy contributions [66,70]. In this respect, various authors [47] have noted that there is little high-quality research in relation to effective interventions with older people in age-friendly environments.

### 4.4. Limitations

Efforts were made to minimise the limitations of this systematic review. However, interpreting results entails the possibility of assuming certain limitations, such as non-inclusion of articles indexed in other literature databases, and potentially losing a significant reference owing to error on the part of the participating researchers. Additionally, in view of the lack of reviews regarding environmental and psychosocial interventions based on the framework of AFCC and active ageing, the decision was taken to use broad inclusion criteria, which favoured the selection of low-quality studies. In fact, the inclusion of uncontrolled interventions increased the proportion of studies evaluated with a high risk of bias. Moreover, the option of performing a meta-analysis was discarded owing to the heterogeneity of the retained references, characterised by diversity of aims, data sources, variables analysed and forms of intervention, the predominance of case studies, and the insufficient information available with which to conduct such an analysis.

## 5. Conclusions

### 5.1. Summary

This systematic review provides a summary of the available empirical evidence in the current academic literature related to the characteristics, content and effectiveness of interventions aimed at improving environmental and psychosocial risk factors for older people, from the perspective of AFCC and the promotion of AA. The results of the review indicate that there have been more non-exhaustive practices than rigorous investigations, with a high proportion of studies showing low methodological quality and a high risk of bias, reflecting a lack of consensus in the literature regarding the conceptual and methodological definition of intervention and AFCC. In fact, there is observed to be limited theoretical understanding and empirical evidence regarding the environmental characteristics and social resources that promote urban age-friendliness and encourage active and healthy ageing. This fact has affected the development of active policies to promote AA. In addition, methodological aspects, such as the absence of random controlled designs and the preponderance of uncontrolled interventions, as well as a lack of more solid longitudinal studies, have made it more difficult to obtain conclusive and reproducible evidence. Of note among the selected studies were multicomponent non-pharmacological interventions, as well as environmental interventions focused on reducing risk and adapting the daily environment. In contrast, psychosocial interventions prioritised social strategies (behavioural changes, promotion of participation) and education.

The assessment of post-intervention effectiveness pointed to a significant improvement in outcomes for AFCC domains such as transportation and housing, followed by behavioural changes to favour the lifestyle of older people, such as increased participation and social inclusion. To the contrary, interventions were linked with reduced effectiveness in health, which would call into question the design of certain interventions and the supposed age-friendliness of some communities, by not promoting physical and psychological health. It is also suggested that the success of interventions that incorporate personal and organisational aspects might have positive effects in the longer term. However, fewer changes would be observed in interventions revolving around changing lifestyles owing to the impact of complex multi-causal factors. In turn, changes associated with the implementation of heterogenous interventions were determined by core components and elements, including: access to information and enhancement of skills; removal of barriers and access to opportunities for choice and social integration; improved quality of services and support, and degree of awareness and social commitment of the various community agents; level of continuity and effectiveness of specific measures and support implemented over time; and finally, degree of involvement of public policies in promoting collaboration between authorities and social agents.

Current research limitations are compromising the implementation and assessment of government programmes. However, the detection of some high-quality studies offers a promising path to facilitate more effective age-friendly interventions aimed at producing key empirical evidence for future public policies on active and healthy ageing. There is also recognition of the difficulty of transferring knowledge of local practices to other, more complex urban and sociocultural systems.

### 5.2. Future Research Lines

According to the obtained results, longitudinal and experimental studies, based on extending the duration of interventions, on one hand, and on the monitoring of participant experiences (fidelity, adherence), on the other hand, are necessary. These types of results would provide new knowledge regarding how environmental changes in age-friendly initiatives affect the experience of ageing in place [128,129].

The changes associated with the implementation of interventions suggest future lines of research focused on: studying the impact of public information campaigns on participation levels among older adults; analysing the degree of impact of the community dynamic on the design, implementation and success of AFCC and AA interventions; considering the complex social causes and governance associated with interventions; evaluating the level of physical, information-related, social and psychological access that interventions offer participants in terms of opportunities for choice and social integration; and examining in greater depth the involvement of the knowledge, awareness and attitudes of the various social agents in the success of interventions aimed at promoting AFCC.

With life expectancy gradually rising, it will be necessary to implement AFCC-related interventions that are focused on advanced-age (nonagenarians and centenarians) and vulnerable groups (disabled, dependent). In this respect, research should be encouraged to determine which sociodemographic groups, measurement instruments and geographical scales to study in order to transfer knowledge regarding the design of future public policies in an ageing society [130].

This review demonstrates the need to encourage new transnational and intercultural research to confirm the effectiveness of interventions in different geographical and sociocultural contexts, and to develop a comprehensive assessment model, in line with other authors [104]. It is also a priority to encourage studies of rural age-friendly communities and attempt to identify the potential implications of degrees of rurality (based on size and proximity to an urban centre) for the effectiveness of age-friendly intervention programmes [131]. Efforts should be made to link studies of setting-based interventions with national policies to adjust to the requirements arising from international documents. The literature has shown the relevance of physical and social environments as a future priority, and their assessment over the course of the years in different geographical spaces can offer key insights as to the actions of states in this regard [2]. Along the same lines, monitoring the 2030 Agenda and the Sustainable Development Goals (SDG) [132] can offer opportunities for future research in terms of assessing AFCC policies [133].

Given the lack of consensus as to the definition of intervention and its multiple associated factors, more theoretical and methodological research is required in the design, implementation, practical implications and evaluation of interventions [26]. This will contribute to producing empirical evidence that will provide knowledge that facilitates the development of public policies to promote AFCC and AA [34,43]. Additionally, the empirical evidence needed for the implementation of interventions with regard to AFCC requires academic mechanisms, such as improved collaboration based on an interdisciplinary approach [7]. Specifically, the interdisciplinary approach of environmental gerontology might facilitate a confluence of researchers from different fields and disciplines, such as health and social sciences, humanities and engineering. There is also a need to promote research intended to strengthen the theoretical foundations and minimise the conceptual problems associated with the AFCC framework. Furthermore, future studies must foster a methodological consensus based on greater use of key indicators, standardised tools and checklists to define the design and implementation of interventions focused on promoting age-friendly communities and active and healthy ageing. All of this is with the purpose of being able to contribute to creating imaginative and inclusive environments, in which older people can continue to be active citizens and the protagonists of their own future and that of their communities.

## Figures and Tables

**Table 1:** Chain of terms used in the search according to databases.

Search in the Web of Science (databases: Core collection; MedLine; Scielo)
((TS = ((Friendl* near (age or ages or aged or aging or ageing or elderly) near (city or cities or communit* or environment* or neighb* or rural or urban*)) or (aged-friend* or age-friend* or ageing-friend* or aging-friend* or elderly-friend*)) and py = 2007–2020)) Refined by: DOCUMENT TYPES (Article OR Review OR Book) AND LANGUAGE (English OR Portuguese OR French OR Spanish)
Search in Scopus
TITLE-ABS-KEY ((aged-friend* OR age-friend* OR ageing-friend* OR aging-friend* OR elderly-friend*) OR (friendl* W/15 (age OR ages OR aged OR aging OR ageing OR elderly) W/15 (city OR cities OR communit* OR environment* OR neighb* OR rural OR urban*))) AND PUBYEAR > 2006 AND (LIMIT-TO (DOCTYPE, “ar”) OR LIMIT-TO (DOCTYPE, “ch”) OR LIMIT-TO (DOCTYPE, “re”) OR LIMIT-TO (DOCTYPE, “bk”)) AND (LIMIT-TO (LANGUAGE, “English”) OR LIMIT-TO (LANGUAGE, “French”) OR LIMIT-TO (LANGUAGE, “Spanish”) OR LIMIT-TO (LANGUAGE, “Portuguese”))

**Table 2:** Charting the data of included studies in the systematic review.

ID.	Authors and Year	Objectives	Geographical Context and Length	Design and Approaches	Participants	Interventions	Instruments and Measures	Effectiveness
[60]	Amoah et al., 2019	To study how conscious and collaborative interventions affect the older persons’ perception of age-friendliness of various AFC domains and the implications for health-related well-being over time	Urban (Hong Kong’s Islands District) 17 months	NC AFCC	NI: 946 PA: ≥50 MA: 71.4% W: 78.1% Randomness: NR	TY: Environmental AP: Risk reduction; environmental adaptations ST: Prevention; management Domain: H; T; OS; CS; SP; RS; CP; IC FI: Individually based	Quantitative methods: pre- and post-intervention study M: self-rated health (Likert scale) DA: Binary logistic regression	HE: with no improvement in self-rated health BE: more participation DE: improvement in 5 domains of AFCC TEE: short term
[61]	Atkins, 2019	To examine how stakeholders (government, peak bodies and the not-for-profit sectors) prioritize age-friendly communities through interventions to improve older people’s well-being	Metropolitan (metropolitan area of Perth, Australia) 2 months	NC AFCC	NI: 117 NG: 23 PA: n/d MA: n/d %W: n/d Randomness: NR	TY: Environmental AP: Risk reduction ST: Prevention Domain: H; T; OS; CS; SP; RS; CP; IC FI: Group format	Quantitative and qualitative methods: Q methodology; semi-structured interviews; focus groups M: n/d DA: Factor analysis	HE: n/d BE: more social participation among older adults DE: ease of use of transport TEE: short term
[62]	Broome et al., 2013	To evaluate the impact of implementing age-friendly guidelines for public buses on bus use, usability and social participation for older persons	Urban (Hervey Bay and North of Brisbane, Queensland, Australia) 24 months	NRC AFCC	N: 335 NI: 100 (users and non-users) PA: ≥60 MA: 72.4 % W:78.9% Randomness: NR	TY: Environmental AP: Risk reduction; socials. ST: Prevention; management Domain: T FI: Individually based	Quantitative methods: pre- and post-intervention study; data were compared with nominal group technique data collected from a previous study M: Social Activities Participation (based on the Social Activities Checklist: SOCACT: frequency and satisfaction scale) DA: Multinomial logistic regression	HE: n/d BE: the frequency of use of transport for older adults Does not increaseDE: improvement the ease of use of transportation TEE: short term
[63]	Gough and Cassidy, 2017	In the context of the Fountain of Health Initiative for Optimal Ageing, related to the AFCC, this paper aimed at assessing the effectiveness of the peer-led educational groups to promote health knowledge and behaviours at the community level	Urban areas (Halifax Regional Municipality) and rural areas (Annapolis Valley, Nova Scotia), Canada 6 weeks (90 min per series)	NRCr AFCC through the Fountain of Health Initiative for Optimal Ageing areas, related to AFCC	Ni: 51 PA: ≥50 MA: n/d %W: 75.3% Randomness: NR	TY: Multicomponent AP: Information and education ST: Management Domain: SP; RS FI: Group format	Quantitative and qualitative methods: pre- and post-intervention study; peer-led education series M: Self-perception of aging based on the Attitudes Towards Own Aging subscale. DA: Chi squared; paired samples *t*-test.	HE: n/d BE: improvement attitudes towards ageing DE: more social participation TEE: short term
[64]	Jitramontree et al., 2015	To develop and implement a Multifactorial Age-friendly Fall Prevention program (MAFPP) among older people living in the community	Intraurban (west of Bangkok, Thailand) Several months (no specification)	NC AFCC Through the Multifactorial Age-friendly Fall Prevention Program (MAFPP)	NI: 50 NF: 20 NP: 10 PA: ≥60 MA: n/d %W: n/d Randomness: NR	TY: Multicomponent AP: Risk reduction; support groups. ST: Prevention; management Domain: H; CP FI: Group format	Qualitative methods: focus groups and in-depth interviews M: Thai Fall Risk Assessment Test (Thai-FRAT) DA: thematic analysis	HE: improvement in the prevention of falls BE: better communication DE: decrease environmental risk in housing TEE: short term
[65]	Kam, 2020	Evaluate the effectiveness of the EPS (Empowerment, Participation and Strengths) intervention model in older users of the public transport system	Intraurban (Hong Kong districts) Several months (no specification)	NC AFCCthrough the EPS principles (Empowerment, Participation and Strength)	N: 1683 NI: 30 PA: ≥60 MA: n/d %W: n/d Randomness: NR	TY: Psychosocial AP: Socials ST: Prevention Domain: T FI: Group format	Quantitative and qualitative methods: survey; focus groups; observational study through site visits; M: structured questionnaireDA: n/d	HE: n/d BE: user satisfaction; empowerment DE: transport service adaptation improvements TEE: short term
[66]	Kuo and Chen, 2019	Under the assumption of the knowledge and attitudes of employees toward ageing are important for successful of Age-Friendly Hospital (AFH), this observational study aimed at examining the certification process of an AFH using John Kotter’s change model and evaluating the changes in employees’ knowledge of ageing and their attitudes towards the elderly	Building (the Cardinal Tien Hospital, Taipei, Taiwan, as a teaching hospital) 8 months	RCr AFCC based on the Age-Friendly Hospital certification process	N: 336 Ni: 163 PA: ≥20 MA: 39.2 %W: n/d Randomness: R	TY: Psychosocial AP: Training ST: Management Domain: CS; RS FI: Individually based and Group format	Quantitative and qualitative methods: self-administered online questionnaire pre- and post-intervention; observational study M: Facts on Ageing Quiz (FAQ1) scale assess physical, psychological and social factors; the Geriatric Attitudes Scale (GAS); change evaluation through SWOT analysis DA: Paired samples *t*-test.	HE: n/d BE: positive effect of employees’ attitude towards ageing DE: improvements in health service SO: improvements in organization and management TEE: short term
[67]	Lee et al., 2018	To evaluate the impact of a community transport intervention on the independence and well-being of older people living in an urban community	Urban (Perth, Australia) 4 months	NC AFCC	NI: 32 (functional disability, no cognitive impairment) PA: ≥65 MA: n/d %W: 75% Randomness: n/d	TY: Environmental AP: Risk reduction; environmental adaptations ST: Prevention Domain: T FI: Individually based	Longitudinal analysis based on quantitative and qualitative methods: pre- and post-intervention interviews, in-depth interviews M: The Personal Component of the Housing Enabler (Functional Limitations and Dependence on Mobility devices) DA: descriptive analyses. Qualitative analysis through NVivo software	HE: no health improvements BE: improves independence and the perception of safety at exits. No communications improvements DE: relative effectiveness of the transport service adaptation improvements TEE: short term
[68]	Levert et al., 2016	This observational analysis aimed at adapting, implementing and evaluating an intervention based on personalized citizen support (Citizen Intervention in Community Living project) for older people with traumatic brain injury (TBI), in order to know the facilitators or the barriers in their use of public spaces in the residential environment.	Intraurban (Montreal, Canadá) 20 h	RCr AFCC	NI: 3 PA: ≥65 MA: 85.3 %W: 66.6% Randomness: R	TY: Multicomponent AP: Risk reduction; socials ST: Prevention Domain: H; T; SP; RS FI: Individually based	Qualitative methods: direct observational study with inductive approach through site visits M: fieldwork script DA: Thematic and cross-sectional analysis	HE: n/d BE: proactive behaviour in exits; preventive social support DE: social participation improvement TEE: short term
[69]	Pejner et al., 2019	To develop and evaluate healthcare technologies through designing, developing and evaluating an age-friendly smart home that uses smart technologies to collect and compile health-related evidence in order to support decision making and communication regarding medication self-managing among older people	Urban (municipalities of Halmstad and Hylte, Halland, Sweden) 3 months (3 h per session)	NRCr AFCC through the Intelligent Age-Friendly Home (IAFH)	NI: 10 participants with polypharmacy and home care NF: 10 NP: 10 PA: ≥65 MA: n/d %W: 69% Randomness: NR	TY: Multicomponent AP: Risk reduction; environmental adaptations; incorporation of assistive technology; training ST: Prevention; management Domain: CS FI: Individually based and group format	Quantitative and qualitative methods: survey; focus groups; participatory design. Several phases: conceptualization of the system; development; pilot study; full-scale intervention M: Morisky Medication Adherence Scale; Personal Well-being Index-Adult; Satisfaction with Life Scale; Serenity Scale DA: n/d	HE: relative effectiveness of adherence to medication BE: relative effectiveness due to an unwilling attitude to the use of assistive technology DE: relative effectiveness of technological systems in the home TEE: short term
[70]	Thompson et al., 2014	To evaluate the effects of residential streets improvements for the support of physical activity and well-being among older adults	Intraurban in locations of England, Wales and Scotland 30 months	NRC AFCC	N: 96 NI: 56 (residents); 40 (non-residents) PA: ≥65 MA: 75.2 %W: 60.4% Randomness: NR	TY: Multicomponent AP: Risk reduction; environmental adaptations; socials ST: Prevention; Management Domain: OS FI: Individually based	Quantitative methods: pre- and post-intervention surveys; Accelerometry. M: general health (EQ-5D) scale; quality of life (CASP-19); frequency of outdoor visits (walking, recreational walking, gardening, outdoor sports, other outdoor activities); Instrumental Activities of Daily Living (IADL); neighbourhood open space (NOS) scale AD: Paired samples *t*-test; factor analysis; hierarchical blocked linear regressions; non-parametric tests (Mann–Whitney U or Kruskal–Wallis)	HE: no health improvements BE: no improvements in activity participation DE: street safety perception improvement TEE: medium term (between 3 and 6 months)

-Col. ID: the same as in the references section. -Col. Design of the intervention and Approaches: NRC: Non-Randomised Controlled intervention; RCr: Randomised Crossover intervention; NRCr: Non-randomised Crossover intervention; NC: Non-Controlled intervention. AFCC Age-Friendly Cities and Communities. -Col. Participants: N: Total participants; NI: Total participants in the intervention; NF: Total family participants; NP: Total professional participants; NG: Total participants of the Government; PA: Age of the participants; MA: Mean age; %W: percentage of women; Randomness: R: Randomized; NR: Non-randomized. -Col. Interventions: TY: Type; AP: Approach; ST: Strategy. Domain of the interventions: H (housing); T (transportation); OS (outdoor spaces and buildings); CS (community support and health services); SP (social participation); RS (respect and social inclusion); CP (civic participation and employment); IC (information and communication). FI: Format of the interventions: single; collective. -Col. Instruments/Measures: M: Measures; DA: Data analysis. -Col. Effectiveness of the interventions: HE: Effectiveness in improving risk factors for physical and psychological health; BE: Effectiveness of behavioural changes in lifestyle; OE: Effectiveness of changes in the results of organizations; DE: Effectiveness of changes in the results of the AFCC domains; TEE: Time elapsed at the end of the intervention for its evaluation. n/d: no data. Source: Own elaboration based on the included studies.

**Table 3:** Effectiveness of interventions in the included studies.

Type of Effectiveness	Significant Changes Reported in the Studies		Success Rate of Interventions									
			Environmental Intervention			Psychosocial Intervention			Multicomponent			Total
	Author/Year	*N*	Studies	SR		Studies	SR		Studies	SR		
			*N*	*N*	%	*N*	*N*	%	*N*	*N*	%	%
Improvement of risk factors for health	Amoah et al., 2019; Atkins, 2019; Gough and Cassidy, 2017; Jitramontree et al., 2015; Lee et al., 2018; Pejner et al., 2019; Thompson et al., 2014	7	3	1	33.3	0	0	0.0	4	2	50.0	42.8
Positive behavioural lifestyle changes	Amoah et al., 2019; Atkins, 2019; Broome et al., 2013; Gough and Cassidy, 2017; Jitramontree et al., 2015; Kam, 2020; Lee et al., 2018; Levert et al., 2016; Pejner et al., 2019; Thompson et al., 2014	10	4	3	75.0	0	0	0.0	5	3	60.0	70.0
Changes in organizational results	Kuo and Chen, 2019	1	0	0	0.0	1	1	100.0	0	0	0.0	100.0
Changes in the results of the Age-Friendly Cities and Communities domains	Amoah et al., 2019; Atkins, 2019; Broome et al., 2013; Gough and Cassidy, 2017; Jitramontree et al., 2015; Kam, 2020; Kuo and Chen, 2019; Lee et al., 2018; Levert et al., 2016; Pejner et al., 2019; Thompson et al., 2014	11	4	2	50.0	2	2	100.0	5	4	80.0	72.7

SR: Success Rate Source: Own elaboration based on the included studies.

**Table 4:** Risk of bias in the included studies.

ID	Authors/Year	Design	Dropout Rates (%)	Risk of Bias				
				Absence of Randomness	Insufficient Information Regarding Participant Characteristics	Insufficient Information Regarding the Instruments and Measures Implemented	Insufficient Information Regarding Results Associated Intervention Effectiveness	Total
				(Degree)	(Degree)	(Degree)	(Degree)	(Degree)
[60]	Amoah et al., 2019	NC	n/d	High	Low	Moderated	Moderated	Moderated
[61]	Atkins, 2019	NC	n/d	High	High	High	High	High
[62]	Broome et al., 2013	NRC	n/d	High	Moderated	Low	Moderated	Moderated
[63]	Gough and Cassidy, 2017	NRCr	52.9	High	Moderated	Moderated	High	High-Moderated
[64]	Jitramontree et al., 2015	NC	n/d	High	High	High	High	High
[65]	Kam, 2020	NC	n/d	Moderated	High	High	High	High-moderated
[66]	Kuo and Chen, 2019	RCr	51.5	Low	Moderated	Low	Moderated	Low-Moderated
[67]	Lee et al., 2018	NC	34.4	High	High	Moderated	Moderated	High-Moderated
[68]	Levert et al., 2016	RCr	0.0	Moderated	Low	High	High	High-moderated
[69]	Pejner et al., 2019	NRCr	n/d	High	Low	High	High	High-moderated
[70]	Thompson et al., 2014	NRC	53.2	Moderated	Low	Low	Low	Low
Total				High	Moderated	Moderated-High	High	High-Moderated

ID: the same as in the references section. n/d: no data. Design of the intervention: NRC: Non-Randomised Controlled intervention; RCr: Randomised Crossover intervention; NRCr: Non-randomised Crossover intervention; NC: Non-Controlled intervention. Source: Own elaboration based on the included studies.
